# Genome Sequence of Sphingobium yanoikuyae B1, a Polycyclic Aromatic Hydrocarbon-Degrading Strain

**DOI:** 10.1128/genomeA.01522-14

**Published:** 2015-02-05

**Authors:** Qiang Zhao, Hongbo Hu, Wei Wang, Huasong Peng, Xuehong Zhang

**Affiliations:** State Key Laboratory of Microbial Metabolism and School of Life Sciences and Biotechnology, Shanghai Jiao Tong University, Shanghai, China

## Abstract

Sphingobium yanoikuyae B1 can utilize biphenyl, naphthalene, phenanthrene, toluene, and m-/p-xylene as sole sources of carbon and energy. Here, we present a 5.2-Mb assembly of its genome. An analysis of the genome can provide insights into the mechanisms of polycyclic aromatic hydrocarbon (PAH) degradation and potentially aid in bioremediation applications.

## GENOME ANNOUNCEMENT

Sphingobium yanoikuyae strain B1, previously known as Sphingomonas yanoikuyae strain B1 and Beijerinckia sp. strain B1 (1), has received much attention due to its versatile capabilities to degrade various pollutants, such as biphenyl, naphthalene, phenanthrene, toluene, m-, p-xylene, anthracene, and so on (2–6). A 40-kb fragment from strain B1 was sequenced, including the alpha and beta subunits of dioxygenase and ferredoxin and ferredoxin reductase involved in the degradation of biphenyl and other polycyclic aromatic hydrocarbons (PAHs) (7, 8). Moreover, the crystal structure of biphenyl 2,3-dioxygenase, the initial dioxygenase for biphenyl and polychlorinated biphenyl degradation, has been modeled in its native and biphenyl bound forms (7). Monocyclic aromatic hydrocarbons like toluene and benzoate are degraded by strain B1 with expression of some *xyl* genes. In addition, strain B1 can degrade polycyclic azaarenes like phenazine by adding two hydroxyl groups on the benzene ring (9). To further investigate the degradation pathways of phenazine, PAHs, and other heterocyclic compounds, we sequenced the genome of strain B1.

DNA was extracted using a Promega Wizard Genomic DNA purification kit, following the manufacturer’s instructions. The draft genome sequence of strain B1 was obtained using an Illumina GAIIx instrument (250-fold coverage) and assembled with Velvet v1.1.07 (10), resulting in 56 scaffolds composed of 111 contigs. The largest contig assembled was approximately 185 kb (the *N*_50_ was approximately 85 kb). The total lengthen of the genome was estimated to be 5,200,045 bp with a G+C content of 64.53%. Putative open reading frames were predicted using Glimmer v3.02 (11). Gene prediction and annotation were carried out using the RAST server and the NCBI PAPPC Pipeline (12, 13). The tRNA genes were predicted using tRNAscan-SE (14). The rRNA genes were analyzed using BLAST with Sphingobium chlorophenolicum L-1 as the template (15). Classification and metabolic pathway analysis of functional genes were determined by KEGG and Clusters of Orthologous Groups (16).

The genome harbors 5,140 putative open reading frames, of which 12 rRNAs and 51 tRNAs were found and 1,206 coding sequences (CDSs) were designated as encoding hypothetical proteins. A total of 35 dioxygenases or putative dioxygenases were found in the genome, including catechol 1, 2-dixoygenase, biphenyl 2,3-dioxygenase, biphenyl-2, 3-diol 1, 2-dioxygenase. In addition, 71 transposase encoding genes were found in the genome, some of which flank the PAH degradation gene cluster and may be related to the dissemination of PAH degradation operons (17). Moreover, 48 ABC transporter-related genes and 82 TonB-dependent receptors were predicted, which may be involved in the PAH transportation (18). The genome sequence of strain B1 provides new insights into the genetic versatility of Sphingobium strains, the mechanism of PAH, and polycyclic azaarenes degradation.

### Nucleotide sequence accession numbers.

This whole-genome shotgun project has been deposited at DDBJ/EMBL/GenBank under the accession no. JPOU00000000. The version described in this paper is version JPOU01000000.
